# The prognostic value of systemic inflammation in patients undergoing surgery for colon cancer: comparison of composite ratios and cumulative scores

**DOI:** 10.1038/s41416-018-0095-9

**Published:** 2018-05-23

**Authors:** Ross D Dolan, Stephen T McSorley, James H Park, David G Watt, Campbell S Roxburgh, Paul G Horgan, Donald C McMillan

**Affiliations:** Academic Unit of Surgery, School of Medicine, Glasgow Royal Infirmary, University of Glasgow, Glasgow, UK

**Keywords:** Surgical oncology, Colon cancer

## Abstract

**Introduction:**

The systemic inflammatory response has been proven to have a prognostic value. There are two methods of assessing the systemic inflammatory response composite ratios (R) and cumulative scores (S). The aim of this study was to compare the prognostic value of ratios and scores in patients undergoing surgery for colon cancer.

**Methods:**

Patients were identified prospectively in a single surgical unit. Preoperative neutrophil (N), lymphocyte (L), monocyte (M) and platelet (P) counts, CRP (C) and albumin (A) levels were recorded. The relationship between composite ratios neutrophil–lymphocyte ratio (NLR), platelet–lymphocyte ratio (PLR), lymphocyte–monocyte ratio (LMR), C-reactive protein albumin ratio (CAR) and the cumulative scores neutrophil– lymphocyte score (NLS), platelet–lymphocyte score (PLS), lymphocyte–monocyte score (LMS), neutrophil– platelet score (NPS), modified Glasgow prognostic score (mGPS) and clinicopathological characteristics, cancer-specific survival (CSS) and overall survival (OS), were examined.

**Results:**

A total of 801 patients were examined. When adjusted for tumour node metastasis (TNM) stage, NLR >5 (*p* < 0.001), NLS (*p* < 0.01), PLS (*p* < 0.001), LMR <2.4 (*p* < 0.001), LMS (*p* < 0.001), NPS (*p* < 0.001), CAR >0.22 (*p* < 0.001) and mGPS (*p* < 0.001) were significantly associated with CSS. In patients undergoing elective surgery (*n* = 689), the majority of the composite ratios/scores correlated with age (*p* < 0.01), BMI (*p* < 0.01), T stage (*p* < 0.01), venous invasion (*p* < 0.01) and peritoneal involvement (*p* < 0.01). When NPS (myeloid) and mGPS (liver) were directly compared, their relationship with CSS and OS was similar.

**Conclusions:**

Both composite ratios and cumulative scores had prognostic value, independent of TNM stage, in patients with colon cancer. However, cumulative scores, based on normal reference ranges, are simpler and more consistent for clinical use.

## Introduction

Colorectal cancer is the fourth most common cancer in the United Kingdom and the second most common cause of cancer death.^1^ Despite death rates from colorectal cancer falling by approximately 14% over the last decade, approximately 40% of those diagnosed will die from their colorectal cancer. Surgery remains the primary modality of cure in these patients and therefore there is a continuing interest in factors that will effectively identify patients at high risk of dying from their disease following potentially curative surgery.

Over the last decade or so it has become clear that markers of the systemic inflammatory response are clinically useful to identify patients at high risk of tumour progression in a variety of common solid tumours, in particular lung and gastrointestinal cancer.^2,3^ These markers of the systemic inflammatory response are usually based around composite ratios or cumulative scores of different circulating white blood cells or acute phase proteins representing the systemic responses of two different organs, lymphoid/myeloid tissue and liver, respectively (Table 1). There have been two main approaches to the formation of these prognostic scores. One approach is to take the ratio of different white blood cells and then apply a prognostic threshold to the ratio such that outcome is effectively stratified. The most repeatedly validated example of this approach is the neutrophil–lymphocyte ratio (NLR) based on the ratio of circulating neutrophil and lymphocyte counts (Table 1).^2,3^ Other validated examples are the platelet–lymphocyte ratio (PLR) based on the ratio of circulating platelet and lymphocyte counts (Table 1) and the lymphocyte–monocyte score (LMR) based on the ratio of circulating lymphocyte and monocyte counts (Table 1).^2,3^ Also, recently a similar approach has been applied to the acute phase proteins, C-reactive protein and albumin, and C-reactive protein albumin ratio (CAR) has been recently validated (Table 1).^2,3^ Although it is clear that the above ratios have prognostic value, a disadvantage of the ratio approach is that, depending on the threshold used, an abnormal ratio may be defined with one or both markers having a normal value.

**Table 1:** Systemic inflammation-based prognostic ratios and scores

Ratio/score	Ratio/score
NLR	
Neutrophil count: lymphocyte count	≤3
Neutrophil count: lymphocyte count	3–5
Neutrophil count: lymphocyte count	>5
NLS	
Neutrophil count ≤7.5 × 10^9^/l and lymphocyte count ≥1.5 × 10^9^/l	0
Neutrophil count >7.5 × 10^9^/l and lymphocyte count ≥1.5 × 10^9^/l	1
Neutrophil count ≤7.5 × 10^9^/l and lymphocyte count <1.5 × 10^9^/l	1
Neutrophil count >7.5 × 10^9^/l and lymphocyte count <1.5 × 10^9^/l	2
PLR	
Platelet count: lymphocyte count	≤150
Platelet count: lymphocyte count	>150
PLS	
Platelet count ≤400 × 10^9^/l and lymphocyte count ≥1.5 × 10^9^/l	0
Platelet count >400 × 10^9^/l and lymphocyte count ≥1.5 × 10^9^/l	1
Platelet count ≤400 × 10^9^/l and lymphocyte count <1.5 × 10^9^/l	1
Platelet count >400 × 10^9^/l and lymphocyte count <1.5 × 10^9^/l	2
LMR	
Lymphocyte count: monocyte count	≥2.40
Lymphocyte count: monocyte count	<2.40
LMS	
Lymphocyte count ≥1.5 × 10^9^/l and monocyte count ≤0.80 × 10^9^/l	0
Lymphocyte count ≥1.5 × 10^9^/l and monocyte count ≤0.80 × 10^9^/l	1
Lymphocyte count <1.5 × 10^9^/l and monocyte count >0.80 × 10^9^/l	1
Lymphocyte count <1.5 × 10^9^/l and monocyte count >0.80 × 10^9^/l	2
NPS	
Neutrophil count ≤7.5 × 10^9^/l and platelet count <400 × 10^9^/l	0
Neutrophil count >7.5 × 10^9^/l and platelet count <400 × 10^9^/l	1
Neutrophil count ≤7.5 × 10^9^/l and platelet count >400 × 10^9^/l	1
Neutrophil count >7.5 × 10^9^/l and platelet count >400 × 10^9^/l	2
CAR	
C-reactive protein: albumin	≤0.22
C-reactive protein: albumin	>0.22
mGPS	
C-reactive protein ≤10 mg/l and albumin ≥35 g/l	0
C-reactive protein >10 mg/l and albumin ≥35 g/l l	1
C-reactive protein >10 mg/l and albumin <35 g/l l	2

*NLR* neutrophil–lymphocyte ratio, *NLS* neutrophil–lymphocyte score, *CAR* C-reactive protein albumin ratio, *mGPS* modified Glasgow prognostic score, *NPS* neutrophil–platelet score, *LMS* lymphocyte–monocyte score, *LMR* lymphocyte–monocyte ratio, *PLR* platelet–lymphocyte ratio, *PLS* platelet–lymphocyte score

A simpler approach is the cumulative prognostic score, where markers of the systemic inflammatory response are defined as normal or as abnormal based on their laboratory reference ranges such that two markers with normal values score lowest and have the best outcomes and two markers with abnormal values score highest and have the poorest outcomes. The most widely validated example of this approach is the Glasgow prognostic score (mGPS) based on the acute phase proteins, C-reactive protein and albumin (Table 1).^2,3^ Also, recently the neutrophil–platelet score (NPS) using neutrophils and platelets has been reported.^4^ Clearly, the cumulative score approach can also be applied to the ratios described above (Table 1), such as NLR (termed neutrophil–lymphocyte score (NLS)), PLR (termed platelet–lymphocyte score (PLS)) and LMR (termed lymphocyte–monocyte score (LMS)).

Therefore, the aim of the present study was to compare the prognostic value of systemic inflammatory markers, in particular that of composite ratios and cumulative scores, in patients undergoing surgery for colon cancer.

## Patients and methods

Patients were identified from a prospectively collected and maintained database of colon cancer resections undertaken in a single surgical unit at the Glasgow Royal Infirmary. Consecutive patients who met the following criteria were included: first, those who had preoperative measurement of serum CRP, albumin and differential blood cell counts within 30 days before surgery; second, those who, on the basis of preoperative abdominal computed tomography and laparotomy findings, were considered to have undergone potentially curative resection for colonic cancer between January 1997 and June 2014. Patients with inflammatory bowel disease-related cancer, who underwent resection with palliative intent or local resection only, or had not had preoperative measurement of CRP or albumin, were excluded.^5^ Tumours were staged using the fifth edition of the tumour node metastasis (TNM) classification, with additional data taken from pathological reports issued after resection.^6^ After surgery, all patients were discussed at a multidisciplinary meeting involving surgeons, oncologists, radiologists and pathologists with special interest in colorectal cancer; patients with stage III or high-risk stage II disease and no significant comorbidities precluding chemotherapy use were offered primarily 5-fluorouracil-based adjuvant chemotherapy on the basis of current guidelines at the time.

Preoperative serum CRP, albumin and differential blood cell counts were recorded prospectively. NLR, PLR, LMR and CAR were all calculated by directly dividing the former by the latter (Table 1). The NLS, PLS, LMS, NPS and mGPS were all constructed using normal reference ranges (Table 1).

Patients were routinely followed up for 5 years after surgery. Date and cause of death were crosschecked with the cancer registration system and the Registrar General (Scotland). Death records were complete until 30 June 2017, which acted as the censor date. Cancer-specific survival (CSS) was measured from the date of surgery until the date of death from recurrent or metastatic colonic cancer. Overall survival (OS) was measured until the date of death from any cause. The West of Scotland Research Ethics Committee approved the study.

### Statistics

The cut-off values for individual ratios were examined using receiver operating characteristic (ROC) curve analyses. The threshold values of such characteristics were based on the most prominent point on the ROC curve for 'sensitivity' and '1-specificity', respectively. The optimal threshold values were defined using the Youden index (maximum (sensitivity + specificity − 1)) and these were compared with published validated values to determine the value used in the subsequent analysis.^7,8^ The area under the ROC curve also was calculated. The relationship between NLR, PLR, LMR, CAR, NLS, PLS, LMS and mGPS and both CSS and OS was assessed using Cox proportional hazards regression to calculate hazard ratios (HRs) and 95% confidence intervals (95% CIs). The relationship between NLR, PLR, LMR, CAR, NLS, PLS, LMS and mGPS and patient clinicopathological characteristics was assessed using Pearson's *χ*^2^ tests. In order to adjust for multiple comparisons during the correlation of composite ratios and cumulative scores and clinicopathological characteristics a *p* value of <0.01 was considered significant. All analyses were performed using SPSS version 22.0 (IBM Corp, Armonk, NY, USA).

## Results

From the prospectively maintained database, 801 patients undergoing potentially curative resection for colon cancer were examined (Table 2a). The majority of patients were over 65 years of age (69%), were male (54%), were overweight or obese (57%) and were American Society of Anaesthesiologists' grade 2 or greater (83%). The majority of patients presented electively (86%), had an open resection (85%) and did not receive adjuvant therapy (75%). The majority of patients had either TNM stage II or III disease (86%) with moderate/well- differentiated tumours (*n* = 703, 89%) and venous invasion (52%). The majority of patients had no margin involvement (95%), peritoneal involvement (72%) or tumour perforation (97%) at the time of resection. On follow-up there were 237 (28%) cancer-related deaths and 437 (52%) deaths overall.

**Table 2a:** The clinicopathological characteristics of patients undergoing surgery for colon cancer (*n* = 801)

Variables	*n* = 801 (%)
Age (years)	
<65	248 (31)
65–74	270 (34)
>75	283 (35)
Sex	
Female	371 (46)
Male	430 (54)
BMI^a^	
Underweight	72 (12)
Normal	190 (31)
Overweight	192 (32)
Obese	153 (25)
ASA grade^b^	
1	97 (17)
2	243 (42)
3	208 (36)
4	29 (5)
Presentation	
Elective	689 (86)
Emergency	112 (14)
Type of surgery	
Open	679 (85)
Laparoscopic	122 (15)
Neoadjuvant therapy^c^	
No	782 (99)
Yes	8 (1)
Adjuvant therapy^d^	
No	574 (75)
Yes	194 (25)
T stage	
1	52 (6)
2	76 (10)
3	418 (52)
4	255 (32)
N stage	
0	507 (63)
1	207 (26)
2	87 (11)
TNM stage	
1	116 (14)
2	391 (49)
3	294 (37)
Differentiation^e^	
Mod/well	709 (89)
Poor	86 (11)
Venous invasion^f^	
No	383 (48)
Yes	416 (52)
Margin involvement^f^	
No	757 (95)
Yes	42 (5)
Peritoneal involvement^f^	
No	578 (72)
Yes	221 (28)
Tumour perforation^f^	
No	772 (97)
Yes	27 (3)

^a^*n* = 607.

^b^*n*  = 575.

^c^*n* = 790.

^d^*n*  = 778.

^e^*n* = 795.

^f^*n* = 799.

*BMI* body mass index, *ASA* American Society of Anaesthesiologists, *TNM* tumour node metastasis

The relationship between the composite ratios and cumulative scores and the clinicopathological characteristics of patients undergoing elective surgery for colon cancer is shown in Table 2b (*n* = 689). There was statistically significant correlation between the majority of the composite ratios and cumulative scores and age (*p* < 0.01), BMI (*p* < 0.01), T stage (*p* < 0.01), venous invasion (*p* < 0.01) and peritoneal involvement (*p* < 0.01).

**Table 2b:** The correlation between composite ratios and cumulative scores and clinicopathological characteristics of patients undergoing elective surgery for colon cancer (*n* = 689)

	Age	Sex	BMI	ASA grade	T stage	N stage	Differentiation	Venous invasion	Margin involvement	Peritoneal involvement	Tumour perforation	Adjuvant therapy
NLR	0.009	0.398	<0.001	0.156	0.069	0.287	0.018	0.002	0.219	0.195	<0.001	0.063
NLS	0.002	0.746	0.003	0.880	0.039	0.504	0.073	0.078	0.069	0.062	0.004	0.301
PLR	<0.001	0.391	<0.001	0.294	0.001	0.395	0.087	0.214	0.095	0.002	0.803	0.758
PLS	0.008	0.827	<0.001	0.337	0.001	0.449	0.029	0.002	0.012	0.005	0.043	0.907
LMR	<0.001	0.004	0.030	0.705	0.063	0.948	0.557	0.133	0.750	0.085	0.041	0.067
LMS	<0.001	0.872	0.165	0.841	0.001	0.412	0.044	0.158	0.033	<0.001	0.184	0.097
NPS	0.649	0.990	0.016	0.753	0.004	0.017	0.005	0.013	0.015	0.277	0.375	0.341
CAR	0.008	0.618	0.027	0.009	<0.001	0.071	0.001	0.011	0.037	0.007	0.004	0.341
mGPS	0.180	0.913	<0.001	0.294	<0.001	0.616	<0.001	0.006	0.005	0.003	0.001	0.422

*p <0.01 is considered significant.

*NLR* neutrophil–lymphocyte ratio, *NLS* neutrophil–lymphocyte score, *CAR* C-reactive protein albumin ratio, *mGPS* modified Glasgow prognostic score, *NPS* neutrophil–platelet score, *LMS* lymphocyte–monocyte score, *LMR* lymphocyte–monocyte ratio, *PLR* platelet–lymphocyte ratio, *PLS* platelet–lymphocyte score

The relationship between composite ratios and cumulative scores and their component values in patients undergoing surgery for colon cancer is shown in Table 2c (*n* = 801). The majority were not assigned as systemically inflamed prior to surgery according to either ratios or scores (NLR >5—19%, NLS >0—47%, PLR >150—65%, PLS >0—48%, NPS >0—28%, CAR >0.22—49%, mGPS >0—41%).

**Table 2c:** The relationship between composite ratios and cumulative scores and their component values in patients undergoing surgery for colon cancer (*n* = 801)

	*n* (%)	Median (range)	Median (range)
		Neutrophil	Lymphocyte
NLR			
≤3	388 (48.4)	4.2 (0.4–9.0)	2.0 (0.7–14.1)
3–5	260 (32.5)	5.5 (2.1–17.5)	1.5 (0.5–4.7)
>5	153 (19.1)	8.5 (2.2–21.3)	1.1 (0.3–2.5)
NLS			
0	421 (52.6)	4.8 (1.7–7.5)	2.0 (1.5–14.1)
1	325 (40.6)	5.1 (0.4–20.6)	1.3 (0.3–4.70)
2	55 (6.9)	9.9 (7.6–21.3)	1.1 (0.5–1.4)
		Platelet	Lymphocyte
PLR^a^			
≤150	237 (34.8)	248 (93–653)	2.1 (1.0–14.1)
>150	445 (65.2)	325 (119–814)	1.40 (0.30–4.70)
PLS^a^			
0	351 (51.5)	282 (94–396)	2.0 (1.5–14.1)
1	283 (41.5)	292 (93–814)	1.3 (0.3–11.0)
2	48 (7.0)	478 (406–698)	1.1 (0.6–1.4)
		Lymphocyte	Monocyte
LMR^b^			
≥2.4	252 (61.0)	1.9 (0.6–14.1)	0.6 (0.1–1.3)
<2.4	161 (39.0)	1.3 (0.3–3.0)	0.8 (0.3–2.0)
LMS^b^			
0	214 (51.8)	2.0 (1.5–14.1)	0.6 (0.1–0.8)
1	169 (40.9)	1.3 (0.3–4.6)	0.7 (0.1–2.0)
2	30 (7.3)	1.2 (0.6–1.4)	1.0 (0.9–1.9)
		Neutrophil	Platelet
NPS^a^			
0	491 (72.0)	4.5 (0.4–7.50)	268 (93–400)
1	140 (20.5)	6.7 (2.3–18.8)	415 (96–811)
2	51 (7.5)	9.8 (7.6–20.60)	474 (406–814)
		CRP	Albumin
CAR			
≤0.22	412 (51.4)	5 (0.1–9)	38 (21–49)
>0.22	389 (48.6)	22 (6–339)	35 (15–47)
mGPS			
0	474 (59.2)	5 (0.1–10)	38 (21–49)
1	173 (21.6)	22 (11–220)	38 (35–47)
2	154 (19.2)	37 (11–339)	31 (15–34)

^a^*n* = 682.

^b^*n* = 413.

*NLR* neutrophil–lymphocyte ratio, *NLS* neutrophil–lymphocyte score, *CAR* C-reactive protein albumin ratio, *mGPS* modified Glasgow prognostic score, *NPS* neutrophil–platelet score, *LMS* lymphocyte–monocyte score, *LMR* lymphocyte–monocyte ratio, *PLR* platelet–lymphocyte ratio, *PLS* platelet–lymphocyte score

The median values for the components of the ratios and scores are shown in Table 2c. An NLR 3–5 was associated with a median neutrophil count of 5.5 × 10^9^/l and a median lymphocyte count of 1.5 × 10^9^/l, both within the normal reference range. In contrast, an NLR >5 was associated with a median neutrophil count of 8.5 × 10^9^/l and a median lymphocyte count of 1.1 × 10^9^/l, both outside the normal reference range. A PLR >150 was associated with a median platelet count of 325 × 10^9^/l and a median lymphocyte count of 1.4 × 10^9^/l, the platelet count being within the normal reference range. An LMR <2.4 was associated with a median lymphocyte count of 1.3 × 10^9^/l and a median monocyte count of 0.8 × 10^9^/l, monocyte count being within the normal reference range. A CAR >0.22 was associated with a median CRP concentration of 24 mg/l and a median albumin concentration of 36 g/l, with albumin being within the normal reference range.

**Fig. 1:**
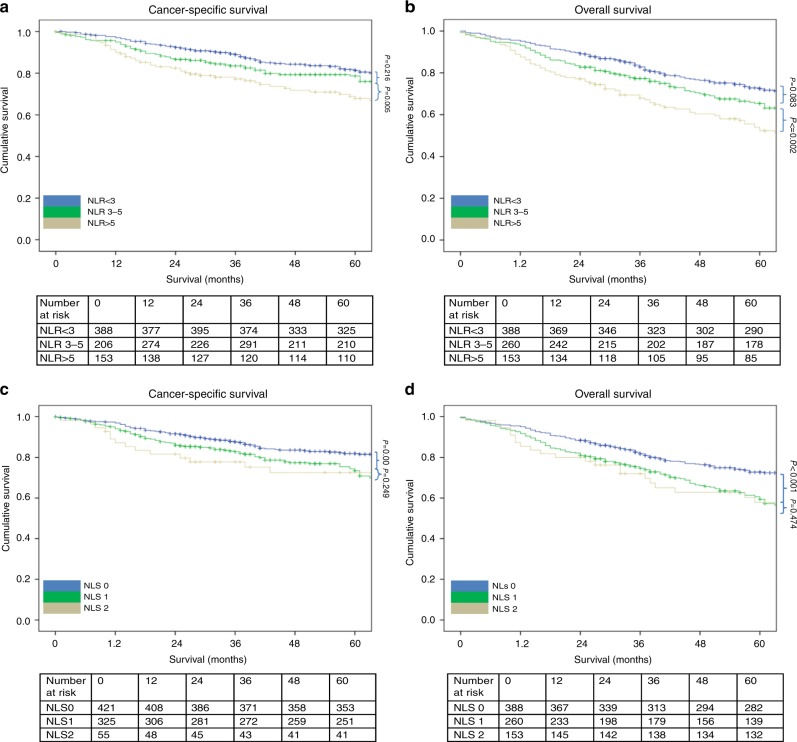
**a–d** The relationship between the NLR and NLS and both CSS and OS in patients undergoing surgery for colon cancer. Number at risk depicts the number of patients alive or not censored entering each time period

**Fig. 2:**
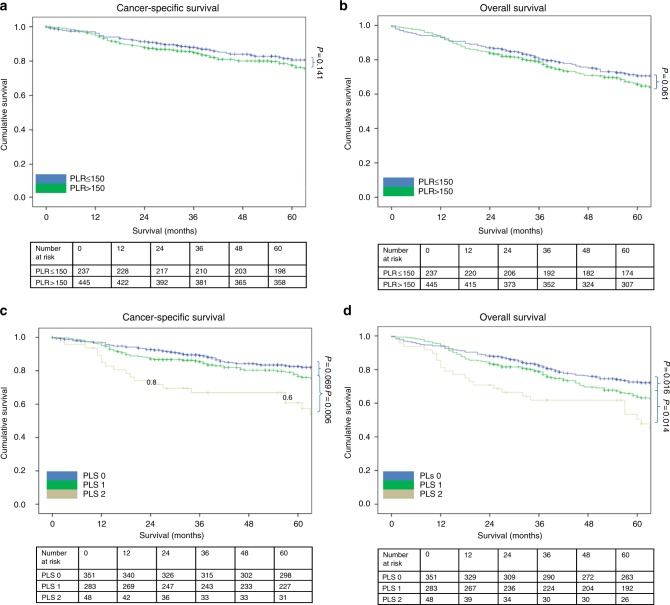
**a–d** The relationship between the PLR and PLS and both CSS and OS in patients undergoing surgery for colon cancer. Number at risk depicts the number of patients alive or not censored entering each time period

**Fig. 3:**
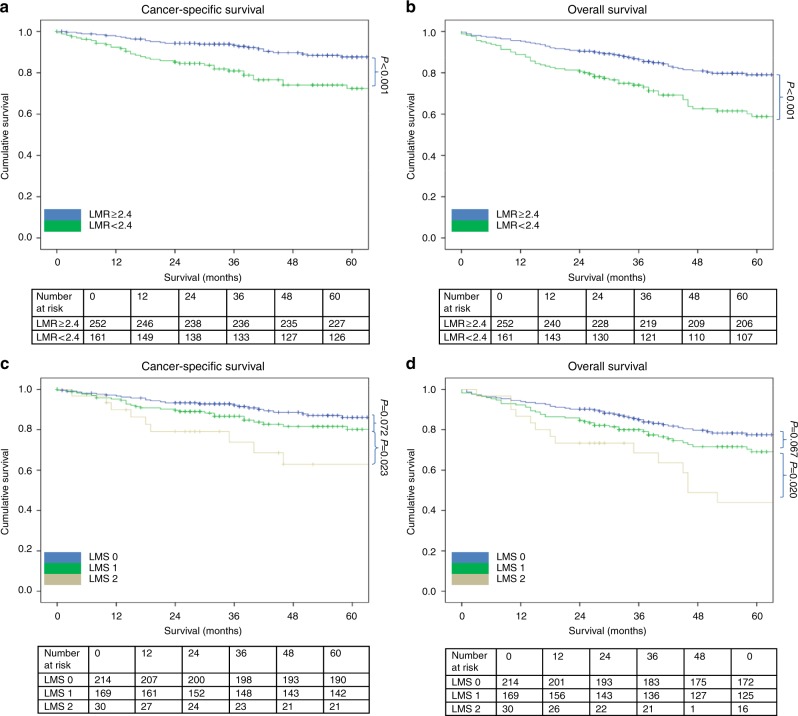
**a–d** The relationship between the LMR and LMS and both CSS and OS in patients undergoing surgery for colon cancer. Number at risk depicts the number of patients alive or not censored entering each time period

**Fig. 4:**
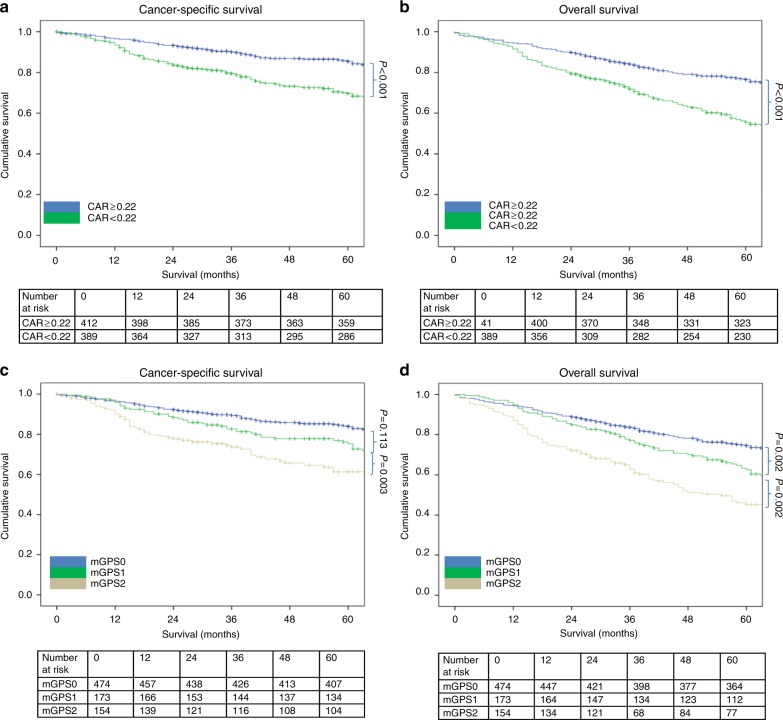
**a–d** The relationship between the CAR and mGPS and both CSS and OS in patients undergoing surgery for colon cancer. Number at risk depicts the number of patients alive or not censored entering each time period

The relationship between validated ratios, scores and 5-year CSS in patients undergoing surgery for colon cancer is shown in Table 3 and Figs. 1–4. On ROC analysis using standard thresholds and CSS as an end-point, the AUC for TNM stage was 0.649, NLR was 0.577, NLS was 0.566, PLR was 0.538, PLS was 0.607, LMR was 0.613, LMS was 0.605, NPS was 0.580, CAR was 0.582 and mGPS was 0.591. When adjusted for TNM stage, NLR >5 (*p* < 0.001), NLS 1 and 2 (both *p* ≤ 0.01), PLS 2 (*p* < 0.001), LMR <2.4 (*p* < 0.001), LMS 2 (*p* < 0.001), NPS 2 (*p* ≤ 0.001), CAR >0.22 (*p* < 0.001), mGPS 2 (*p* < 0.001) were significantly associated with CSS.

**Table 3:** The relationship between validated ratios, scores and survival in patients undergoing surgery for colon cancer (*n* = 801)

	Univariate			Multivariate Adjusted for TNM stage		Univariate			Multivariate Adjusted for TNM stage	
	AUC (95% CI)	CSS HR (95% CI)	*p* value	CSS HR (95% CI)	*p* value	AUC (95% CI)	OS HR (95% CI)	*p* value	OS HR (95% CI)	*p* value
TNM stage										
I (*n* = 116)	0.649 (0.559–0.740)					0.569 (0.477–0.661)				
II (*n* = 391)		4.39 (1.78–10.85)	0.001				1.73 (1.16–2.57)	0.007		
III (*n* = 294)		9.86 (4.02–24.17)	<0.001				2.54 (1.70–3.79)	<0.001		
NLR/NLS										
NLR <3 (*n* = 388)	0.577 (0.529–0.624)					0.594 (0.554–0.633)				
NLR 3–5 (*n* = 260)		1.22 (0.87–1.72)	0.251	1.28 (0.91–1.80)	0.152		1.21 (0.95–1.53)	0.118	1.26 (0.99–1.59)	0.061
NLR >5 (*n* = 153)		2.06 (1.46–2.92)	<0.001	2.11 (1.50–3.00)	<0.001		1.85 (1.44–2.37)	<0.001	1.88 (1.46–2.42)	<0.001
NLS 0 (*n* = 421)	0.566 (0.519–0.613)					0.586 (0.546–0.626)				
NLS 1 (*n* = 325)		1.49 (1.10–2.01)	0.010	1.57 (1.16–2.12)	0.003		1.45 (1.17–1.79)	0.001	1.49 (1.21–1.85)	<0.001
NLS 2 (*n* = 55)		2.01 (1.22–3.30)	0.006	1.85 (1.12–3.05)	0.016		1.68 (1.15–2.46)	0.007	1.59 (1.09–2.33)	0.016
PLR/PLS^a^										
PLR ≤150 (*n* = 237)	0.538 (0.486–0.589)					0.555 (0.512–0.598)				
PLR >150 (*n* = 445)		1.31 (0.92–1.86)	0.141	1.20 (0.84–1.70)	0.326		1.26 (0.98–1.63)	0.073	1.20 (0.93–1.55)	0.166
PLS 0 (*n* = 351)	0.578 (0.525–0.631)					0.586 (0.542–0.629)				
PLS 1 (*n* = 283)		1.39 (0.98–1.96)	0.061	1.33 (0.94–1.88)	0.106		1.34 (1.05–1.70)	0.020	1.29 (1.01–1.65)	0.040
PLS 2 (*n* = 48)		2.77 (1.67–4.59)	<0.001	2.42 (1.46–4.01)	0.001		2.16 (1.46–3.18)	<0.001	1.94 (1.31–2.87)	0.001
LMR/LMS^b^										
LMR ≥2.4 (*n* = 161)	0.613 (0.539–0.688)					0.590 (0.528–0.652)				
LMR <2.4 (*n* = 252)		2.62 (1.61–4.27)	<0.001	2.49 (1.53–4.06)	<0.001		2.08 (1.44–3.00)	<0.001	1.99 (1.38–2.87)	<0.001
LMS 0 (*n* = 214)	0.605 (0.528–0.681)					0.585 (0.522–0.648)				
LMS 1 (*n* = 169)		1.69 (0.99–2.86)	0.051	1.65 (0.97–2.81)	0.064		1.47 (0.99–2.17)	0.058	1.41 (0.95–2.10)	0.088
LMS 2 (*n* = 30)		3.68 (1.81–7.49)	<0.001	3.67 (1.80–7.49)	<0.001		2.81 (1.59–4.95)	<0.001	2.76 (1.56–4.88)	<0.001
NPS^a^										
NPS 0 (*n* = 491)	0.580 (0.526–0.634)					0.576 (0.532–0.619)				
NPS 1 (*n* = 140)		1.76 (1.22–2.55)	0.003	1.47 (1.02–2.13)	0.042		1.64 (1.26–2.14)	<0.001	1.47 (1.12–1.92)	0.005
NPS 2 (*n* = 51)		2.50 (1.52–4.10)	<0.001	2.14 (1.30–3.51)	0.003		1.83 (1.24–2.70)	0.002	1.65 (1.12–2.44)	0.011
CAR/mGPS										
CAR ≤0.22 (*n* = 412)	0.582 (0.536–0.628)					0.603 (0.563–0.642)				
CAR >0.22 (*n* = 389)		1.88 (1.40–2.51)	<0.001	1.76 (1.31–2.35)	<0.001		1.88 (1.53–2.31)	<0.001	1.84 (1.49–2.26)	<0.001
mGPS 0 (*n* = 474)	0.591 (0.544–0.639)					0.623 (0.582–0.663)				
mGPS 1 (*n* = 173)		1.35 (0.95–1.94)	0.099	1.22 (0.85–1.75)	0.282		1.49 (1.17–1.90)	0.001	1.44 (1.12–1.84)	0.004
mGPS 2 (*n* = 154)		2.47 (1.77–3.46)	<0.001	2.31 (1.65–3.25)	<0.001		2.32 (1.81–2.99)	<0.001	2.28 (1.76–2.95)	<0.001

^a^*n* = 682.

^b^*n* = 413.

*AUC* area under the curve, *CI* confidence interval, *HR* hazard ratio, *CSS* cancer-specific survival, *OS* overall survival, *TNM* tumour node metastasis, *NLR* neutrophil–lymphocyte ratio, *NLS* neutrophil–lymphocyte score, *CAR* C-reactive protein albumin ratio, *mGPS* modified Glasgow prognostic score, *NPS* neutrophil–platelet score, *LMS* lymphocyte–monocyte score, *LMR* lymphocyte–monocyte ratio, *PLR* platelet–lymphocyte ratio, *PLS* platelet–lymphocyte score

On ROC analysis using standard thresholds and 5-year OS as an end-point, the following AUC for TNM stage was 0.569, NLR was 0.594, NLS was 0.586, PLR was 0.555, PLS was 0.620, LMR was 0.590, LMS was 0.585, NPS was 0.576, CAR was 0.603 and mGPS was 0.623. When adjusted for TNM stage, NLR >5 (*p* < 0.001), NLS 1 and 2 (both *p* ≤ 0.01), PLS 2 (*p* < 0.001), LMR <2.4 (*p* < 0.001), LMS 2 (*p* < 0.001), NPS 2 (*p* ≤ 0.01), CAR >0.22 (*p* < 0.001), mGPS 2 (*p* < 0.001) were all significantly associated with overall survival (Table 3 and Figs. 1–4).

The complementary prognostic value of the cumulative scores NPS and mGPS, markers of innate immune activation from two different organs, were examined in the context of TNM staging (Table 4). Within TNM stage II disease the 5-year CSS rate was 82% and the 5-year CSS rate varied between 86 and 73% according to the NPS and between 86 and 79% according to the mGPS. The 5-year OS rate was 57% and the 5-year OS rate varied between 61 and 47% according to the NPS and between 65 and 48% according to the mGPS.

**Table 4:** The relationship between mGPS, NLS and 5-year CSS and OS rates in patients undergoing potentially curative resection of TNM stage II (*n* = 391) and III (*n* = 294) colonic cancer

	Stage II (*n* = 322)						Stage II (*n* = 322)					
	mGPS 0		mGPS 1/2			mGPS 0–2	mGPS 0		mGPS 1/2			mGPS 0–2
	*n*	5-year CSS (%)	*n*	5-year CSS (%)	*n*		*n*	5-year OS (%)	*n*	5-year OS (%)	*n*	
NPS 0	147 (85%)	88.4 (0.03)	78 (52%)	82.1 (0.04)	**225**	**86.2 (0.02)**	147 (85%)	66.7 (0.04)	78 (52%)	58.7 (0.06)	**225**	**61.3 (0.03)**
NPS 1/2	26 (15%)	69.2 (0.09)	71 (48%)	74.6 (0.05)	**97**	**73.2 (0.05)**	26 (15%)	57.7 (0.10)	71 (48%)	43.7 (0.06)	**97**	**47.4 (0.05)**
NPS 0–2	**173**	**85.5 (0.03)**	**149**	**78.5 (0.03)**	**322**	**82.3 (0.02)**	**173**	**65.3 (0.04)**	**149**	**47.7 (0.04)**	**322**	**57.1 (0.03)**

	Stage III (*n* = 254)						Stage III (*n* = 254)					
NPS 0	120 (82%)	70.0 (0.04)	50 (46%)	60.0 (0.07)	**170**	**67.1 (0.04)**	120 (82%)	54.2 (0.05)	50 (46%)	44.0 (0.07)	**170**	**51.2 (0.04)**
NPS 1/2	25 (18%)	64.0 (0.10)	59 (54%)	57.6 (0.07)	**84**	**59.5 (0.05)**	25 (18%)	48.0 (0.10)	59 (54%)	32.2 (0.06)	**84**	**36.9 (0.05)**
NPS 0–2	**145**	**69.0 (0.04)**	**109**	**58.7 (0.05)**	**254**	**64.6 (0.03)**	**145**	**53.1 (0.04)**	**109**	**37.6 (0.05)**	**254**	**46.5 (0.03)**

Values are expressed as % (standard error) survival not calculated if *n* < 10.

*CSS* cancer-specific survival, *OS* overall survival, *TNM* tumour node metastasis, *NLS* neutrophil–lymphocyte score, *mGPS* modified Glasgow prognostic score, *NPS* neutrophil–platelet score

Within TNM stage III disease, the 5-year CSS rate was 65% and the 5-year CSS rate varied between 67 and 60% according to the NPS and between 69 and 59% according to the mGPS. The 5-year OS rate was 47% and the 5-year OS varied between 51 and 37% according to the NPS and between 53 and 38% according to the mGPS (Table 4).

## Discussion

The results of the present study directly compare, for the first time, the prognostic value of composite ratios and cumulative scores of the systemic inflammatory response. These ratios and scores, whether composed of white cells from lymphoid/myeloid tissue or from acute phase proteins from the liver, had prognostic value, independent of TNM stage, in patients with colon cancer. Moreover, systemic inflammation scores from different organs had similar prognostic value. Taken together, the systemic inflammatory response represents an important prognostic domain to be monitored in patients with colon cancer.

**Fig. 5:**
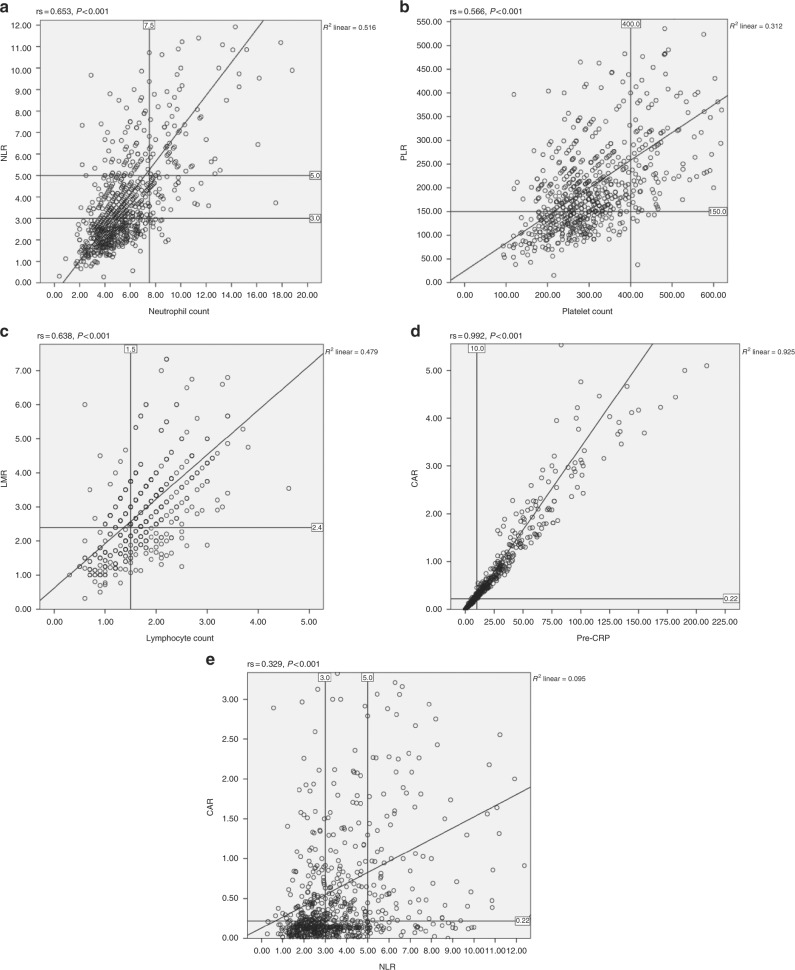
**a–e** Plot of preoperative neutrophil count and NLR, platelet count and PLR, lymphocyte count and LMR, CRP and CAR, NLR and CAR in all patients undergoing surgical resection for colon cancer

In the present study, it was of interest that the ratio thresholds did not always differentiate normal from abnormal values of the composite values. The discrepancy between the ratio threshold and the abnormal single component is shown in Fig. 5a–e. In Fig. 5a, using the line of best fit, an NLR >5 was associated with a median neutrophil count of approximately 7.5, at the top of the normal reference range. In contrast, a NLR >3 was associated with a neutrophil count of approximately 4.5, within in the normal reference range. With reference to PLR >150, it was associated with a platelet count of approximately 200, within the normal range (Fig. 5b). With reference to LMR <2.4, it was associated with a lymphocyte count of 1.5, at the bottom of the normal range (Fig. 5c). Finally, with reference to CAR >0.22 was associated with a CRP of 10 well above the normal range (Fig. 5d). Therefore, it is clear that a number of ratios (e.g. NLR >3 and PLR >150) do not describe components with abnormal values. Moreover, the ratios, compared with scores, consistently assigned a higher proportion of patients to be systemically inflamed. Given that scores based on abnormal value are simpler to construct and have similar and overlapping prognostic value, independent of TNM stage, compared with composite ratios (Table 3), the rationale for the continued use of such ratios is problematic. Indeed, recent clinical calculators for survival in patients with metastatic colorectal cancer, based on data of more than 20,000 patients from randomised controlled trials (ARCAD database), has incorporated the white cell count, neutrophil count, platelet count and albumin level as scores rather than derived ratios.^9,10^ Furthermore Dupré and Malik^11^ have argued that the variability of reported prognostic thresholds of NLR, PLR and LMR questions their reliability for routine clinical practice.

Although it is presumed that composite ratios of lymphoid/myeloid cells and acute phase proteins reflect similar aspects of the systemic inflammatory response, it is clear from the plot of NLR and CAR (Fig. 5e) that these ratios do not simply mirror one another. In contrast, when cumulative scores such as NPS and mGPS, based on normal reference ranges, were compared there was better agreement in terms of systemic inflammatory response status and prognostic value (Table 4). However, it should be noted that although C-reactive protein and albumin are similar proteins components of a differential WCC such as neutrophil count are composed of a number of cell types.^12^ Irrespective the cumulative score approach, based on normal reference ranges, improves our understanding of aspects of the activation of the innate systemic inflammatory response. The simplicity and consistency of this approach has much to commend it.

The innate systemic inflammatory response in patients with cancer, as well as incorporating responses from lymphoid/myeloid tissue and the liver, incorporates responses from other organs and tissues. In particular, the response from the sympathetic nervous system is of interest since similar to that of NPS and mGPS, it is intimately connected with immune responses.^13^ Having established, in patients with cancer, the prognostic value of simple and objective markers of activation of lymphoid/myeloid and liver tissue activation, it would be of considerable interest to examine the prognostic value of objective markers of activation of the sympathetic nervous system.

In the present study, there was a clear correlation between higher composite ratios and cumulative scores and increased age, BMI, advanced T stage and the presence of both venous and peritoneal invasion. These clinicopathological characteristics are also directly associated with a poorer prognosis adding further weight to the prognostic ability of both composite ratios and cumulative score in patients with colonic cancer.

Recently, Park et al.^5^ reported that the mGPS provides complimentary prognostic information to current TNM-based staging. When TNM staging and mGPS were combined, the 5-year OS ranged from 92% (TNM 0, mGPS = 0) to 26% (stage III, mGPS = 2) and the 10-year OS ranged from 92% (TNM 0, mGPS = 0) to 17% (TNM III, mGPS = 2) (*p* < 0.001). This further highlights the prognostic ability of the mGPS which is complementary to the gold standard of TNM staging with both being routinely available worldwide.^5^

The present study has a number of possible limitations. Although a relatively large prospective cohort, there were small numbers of observations in some sub-group analysis. Furthermore, data relating to other factors that may have affected markers of the systemic inflammatory response such drugs taken prior to sampling were not available. Although the present study used the 5th rather than the 7th edition of the TNM staging system, this was recommended in the 2014 Colorectal Cancer Care Guidelines of the Royal College of Pathologists and as such is the basis for all current UK wide practice.^14^ Furthermore, migration from the 5th to 7th edition would be expected to account for an upstaging from node-negative to node-positive disease in <3% of cases, with little subsequent effect on prognosis.^14–16^

A maximum of a 30-day interval between laboratory testing and surgery may be considered to be too long. However, this timescale has been widely reported in the literature and consistent with the chronic nature of the standardised incidence ratio in patients with cancer.^3^ Also, patients with inflammatory bowel disease-related cancers were not included in the analysis. As such, the patient confounding factors of active systemic inflammatory disease and acute changes in the inflammatory state have been minimised.

In summary, present study directly compares, for the first time, the prognostic value of composite ratios and cumulative scores of the systemic inflammatory response. These ratios and scores, whether composed of white cells from lymphoid/myeloid tissue or from acute phase proteins from the liver, had prognostic value, independent of TNM stage, in patients with colon cancer. However, cumulative scores, based on normal reference ranges, are simpler and more consistent for clinical use.
